# Report from a multidisciplinary meeting on anxiety as a non-motor manifestation of Parkinson’s disease

**DOI:** 10.1038/s41531-019-0102-8

**Published:** 2019-12-11

**Authors:** Gregory M. Pontone, Nadeeka Dissanayaka, Liana Apostolova, Richard G. Brown, Roseanne Dobkin, Kathy Dujardin, Joseph H. Friedman, Albert F. G. Leentjens, Eric J. Lenze, Laura Marsh, Lynda Mari, Oury Monchi, Irene H. Richard, Anette Schrag, Antonio P. Strafella, Beth Vernaleo, Daniel Weintraub, Zoltan Mari

**Affiliations:** 1 Department of Psychiatry and Behavioral Sciences, Johns Hopkins University School of Medicine, Baltimore, MD USA; 2 Department of Neurology, Johns Hopkins University School of Medicine, Baltimore, MD USA; 3 The University of Queensland Centre for Clinical Research, Faculty of Medicine, Brisbane, Australia; 4 School of Psychology, The University of Queensland, Brisbane, Australia; 5 Department of Neurology, Royal Brisbane & Woman’s Hospital, Brisbane, Australia; 6 Department of Neurology, Indiana University School of Medicine, Indianapolis, IN USA; 7 Department of Psychology, Institute of Psychiatry Psychology and Neuroscience, King’s College London, London, UK; 8 South London and Maudsley NHS Foundation Trust, London, UK; 9 Department of Psychiatry, Rutgers University, Robert Wood Johnson Medical School, Piscataway, NJ USA; 10 Department of Neurology and Movement Disorders, Lille University Medical Center, Lille, France; 11 Movement Disorders Program, Butler Hospital; Department of Neurology, Warren Alpert Medical School of Brown University, Providence, RI USA; 12 Department of Psychiatry, Maastricht University Medical Center, Maastricht, the Netherlands; 13 Department of Psychiatry, Washington University School of Medicine, St. Louis, MO USA; 14 Michael E. DeBakey Veterans Affairs Medical Center, Houston, TX USA; 15 Department of Psychiatry, Baylor College of Medicine, Houston, TX USA; 16 Person Holistic Innovation, Las Vegas, NV USA; 17 Departments of Clinical Neurosciences and Radiology, Hotchkiss Brain Institute, University of Calgary, Calgary, Canada; 18 Department of Neurology, University of Rochester Medical Center, Rochester, NY USA; 19 Department of Clinical and Movement Neurosciences, University College London, London, UK; 20 E.J. Safra Parkinson Disease Program, Toronto Western Hospital & Krembil Research Institute, UHN; Research Imaging Centre, Campbell Family Mental Health Research Institute, CAMH; University of Toronto, Ontario, Canada; 21 Parkinson’s Foundation, New York, NY USA; 22 Departments of Psychiatry and Neurology, Perelman School of Medicine at the University of Pennsylvania, Philadelphia, PA USA; 23 Parkinson’s Disease Research, Education and Clinical Center, Philadelphia Veterans Affairs Medical Center, Philadelphia, PA USA; 24 Cleveland Clinic Lou Ruvo Center for Brain Health, Movement Disorders Program, Las Vegas, NV USA

**Keywords:** Parkinson's disease, Parkinson's disease

## Abstract

Anxiety is a severe problem for at least one-third of people living with Parkinson’s disease (PD). Anxiety appears to have a greater adverse impact on quality of life than motor impairment. Despite its high prevalence and impact on daily life, anxiety is often undiagnosed and untreated. To better address anxiety in PD, future research must improve knowledge about the mechanism of anxiety in PD and address the lack of empirical evidence from clinical trials. In response to these challenges, the Parkinson’s Foundation sponsored an expert meeting on anxiety on June 13th and 14th 2018. This paper summarizes the findings from that meeting informed by a review of the existing literature and discussions among patients, caregivers, and an international, clinician-scientist, expert panel working group. The goal is to provide recommendations to improve our understanding and treatment of anxiety in PD.

## Introduction

Anxiety occurs in at least one-third of all people with Parkinson’s disease (PWP) and is one of the main determinants of quality of life.^1,2^ In 2016, at the World Parkinson Congress in Portland, Oregon, the Parkinson’s Foundation surveyed Congress attendees with Parkinson’s disease (PD) and their care partners regarding their unmet needs. The survey was part of the Foundation’s Community Choice Research Award initiative, which aims to identify unmet needs within the PD community and address those needs through targeted workshops and research funding. Respondents were given 33 choices and were asked to rank their top 3 unmet needs. Of the 1026 responses received, 141 (13.7%) ranked anxiety as their top unmet need, and 434 (42.3%) ranked anxiety as one of their top three unmet needs. The prominence of anxiety as an unmet need echoes findings from the Parkinson’s UK earlier priority-setting exercise. Using an iterative modified nominal group technique, 1000 individuals, including those with PD, caregivers, family and friends, and healthcare and social service professionals, developed a list of unmet research areas. A subsequent stakeholder consensus meeting identified the top 10 management research priorities; stress and anxiety ranked second after balance and falls.^3^

As a result of the 2016 survey, the Parkinson’s Foundation organized a workshop focused on anxiety in PD in order to discuss gaps in knowledge for both research and care and propose plans for moving the field forward. The June, 2018 workshop participants comprised 16 scientists and clinicians with expertise in PD, including movement disorder neurologists, psychiatrists, psychologists, and PD researchers. This provided an opportunity for specialists from different disciplines to learn how each approached the clinical problem. To provide perspective from the patient side, six members of the PD community attended. These included a family of three (father with Parkinson’s, mother care partner, and adult son) two patients, and a spouse. When asked to share their experience, the family said:

“I was diagnosed with Parkinson’s 15 years ago when I was 43 years old. Before my diagnosis, I was a very active husband, father, son, trial lawyer, and musician. I was easygoing and not anxious about anything! Anxiety makes me feel indecisive and worsens my PD symptoms, particularly slowness and rigidity, tremor, dyskinesia, and body temperature. Anxiety produces unwarranted fear of simple things in life such as how to walk through a room, enter an elevator, airplane or train, choose a meal from the refrigerator, or select my clothing… I think it is essential for the PD community to consider anxiety and emotional needs at the same level as the motor functions since they are so intertwined and not addressing one can interfere with other interventions that might work otherwise.”

- Len Schwartz, person with Parkinson’s

“Anxiety has pervaded every aspect of our lives, even more so than the physical manifestations of PD. It has limited our ability to interact with family and friends, entertain, shop, travel, visit public spaces and try new things. While the physical symptoms can be bad enough on their own, anxiety exacerbates them exponentially.”

-Sharon Aldouby, wife and care partner

“As a son of someone with Parkinson’s, I've always tried to understand the mental toll on my father from this disease. Knowing the difference between what's being caused by the PD and my dad’s actual mindset is essential for me to understand his needs and help him stay grounded.”

-Matt Schwartz, adult son of a person with Parkinson’s

Another attendee wrote:

“*Anxiety turns my mind to jello. It causes me to think in unproductive ways. It destroys my sense of self -worth and renders me almost incapable of making simple choices*.”

-Person with Parkinson’s

Anxiety can dramatically impact many aspects of a person’s life and needs to be addressed in order to improve quality of life for PWP, their family members, and care partners. This review describes the current state of knowledge on anxiety in PD with regard to its presentation and risk factors, clinical approaches to measurement of anxiety, available pharmacological and non-pharmacological therapies, approaches for effective multidisciplinary care, and strategies that help caregivers cope with the increased burden of anxiety in their loved ones.

Anxiety can be assessed as a clinical symptom or a clinical syndrome, with advantages and limitations to each approach. Typically, symptoms can be identified quickly using standardized scales, and specialized training is not required. By contrast, diagnosis of a syndrome, a collection of signs and symptoms that occur together and are usually thought to have a central or shared cause, is more time consuming and requires the synthesis of information, usually requiring specialized training. To that end, syndromes are more reliably associated with an underlying mechanism as compared to symptoms. For example, cough, as a symptom, might be explained by a number of causes, e.g., allergies, the common cold, or tuberculosis, whereas the syndrome of a cough accompanied by bloody sputum, weight loss, fatigue, fever, chills, and chest pain, offers important clues about etiology. Similarly, assessing anxiety in PD as a syndrome is more likely to advance knowledge of underlying mechanisms.

A systematic review of 45 articles published up to September 2015 describing anxiety in a total of 2399 patients found that the point prevalence of anxiety disorders in PD is 31%.^1^ The studies were conducted in countries in Asia, Australia, Europe, North and South America and most—39 of 45—were conducted in outpatient and community settings making this a reasonable estimate of the global prevalence of anxiety in PD. The review found that the most common diagnoses were generalized anxiety disorder (GAD) 14.1%, social phobia 13.8%, and anxiety disorder not otherwise specified (NOS) 13.3%. As a third of patients (31.1%) met criteria for two or more anxiety disorders, the authors raised questions about the construct validity of the Diagnostic and Statistical Manual (DSM) anxiety classifications when applied to PWP. The incidence of anxiety disorders in PD is not known. Studies attempting to determine incidence will first have to address the question of when PD starts, as numerous studies show that anxiety is often present before motor symptoms are detected.^4–6^ Genes associated with PD, a yet to be identified biomarker, or prodromal conditions such as idiopathic RBD may be useful in determining when the association between PD and anxiety begins.

The point prevalence of anxiety disorders in PD is generally thought to be higher than in non-PD populations. Reports on anxiety rates across different populations are problematic because anxiety is frequently reported as a symptom, versus at the level of a syndrome, and studies rarely control for overall disability level. In the few studies with direct comparisons of anxiety rates, findings often differ by anxiety disorder type.^7–9^ For example, in one of the first studies on this issue, Stein MB et al. found greater prevalence of panic disorder and social phobia in PD as compared to multiple sclerosis, type I diabetes, and rheumatoid arthritis, after controlling for disease-related disability.^8^ In a comparison of lifetime prevalence of anxiety in PD to that in dystonia patients and healthy controls from the Epidemiologic Catchment Area study, Lauterbach et al.^9^ found, after controlling for age, gender, and race, higher rates of GAD and social phobia in dystonia compared to PD, and higher rates of simple phobia in PD than in dystonia. Knowledge of the relative prevalence of anxiety syndromes in PD and other neurodegenerative and neurological diseases may provide clarity regarding shared or unique phenomena and focus investigations of underlying mechanisms and treatment.

In PD, occurrence of clinical anxiety syndromes that are unique to PD may also point to unique underlying mechanisms. Several studies have captured these potentially ‘PD-specific’ anxiety disorders using the DSM diagnostic category ‘Anxiety disorder NOS’.^10–13^ Common presentations of ‘NOS’-type anxiety include: a ‘panic-like’ disorder that does not meet DSM criteria for avoidance behavior between episodes, episodic anxiety associated with wearing off of dopaminergic medications, and an anticipatory anxiety in which the individual becomes distressed in advance of and often avoids events in the future. Anxiety in PD can often involve symptoms related to thermoregulation, hypotension, hyperventilation, and trembling, which may represent phenomenological overlap with autonomic dysfunction and other PD-related somatic symptoms.^10^ Research on these apparent ‘PD-specific’ anxiety syndromes is needed to determine the significance of their unusual presentations and to facilitate their diagnosis and clinical management. An alternative explanation or conceptualization of ‘PD-specific’ anxiety is that while the circuity involved in the production of anxiety is universal, the way PD pathology compromises the circuit may be different than the way ‘idiopathic’ anxiety disorders are generated or may alter the clinical expression of symptoms.

The more recent PROMS-PD study^14^ overcame the limitations of many previous studies, which were based on a single assessment of anxiety in relatively small, convenience samples of PWP. This prospective, multicenter, longitudinal study assessed a cohort of 513 PWP annually for up to 4 years. A data-driven analysis identified four reliable classes to which each person could be assigned. At each time point 54–59% of the participants fell into a class labeled ‘Psychologically Healthy’ with a very low probability of any significant anxiety or depression related symptom. The remaining three classes were all associated with significant psychological distress, characterized by varying probabilities of significant anxiety-related symptoms, with or without co-occurring depressive symptoms. Within the distress classes, the anxiety-related profiles revealed were dominated by tension and restlessness, worry, generalized anxiety/panic, irritability, fatigue, slow thinking and poor concentration. Although formal diagnoses were not assigned, such a profile is most consistent with GAD.

The PROMS-PD study also provided the ability to track the pattern of stability or transition of individuals between classes. Overall, 51% were classified as ‘remaining healthy’ throughout the study, with only 8.6% of those in the ‘healthy’ class at baseline transitioning into a ‘distress class’ subsequently. Conversely, the majority of those in one of the three distress-related classes remained in one of those classes between assessments, with 34% ‘remaining distressed’ throughout, and only 6.5% transitioning from a distress group into the Psychologically Healthy group, despite many reporting receiving treatment for their anxiety/depression.

This study reveals a number of important findings. First, while depressive symptomatology was relatively common in the cohort, the anxiety-related symptoms were far more common. Second, the data-driven approach failed to identify a class of patients with prominent depressive symptomatology without co-occurring anxiety symptoms, while the opposite pattern was observed. Third, over time patients transitioned between the mixed depression and anxiety symptoms profile and anxiety dominant profile. Fourth, psychological distress (anxiety +/− depressive symptoms) remained chronic, despite the fact that many individuals were receiving treatment. Finally, even in the most severe group, 40–60% reported receiving no treatment of their anxiety or depression at any time point.

Only a few studies have specifically explored the risk factors for anxiety in PD identifying both PD-specific and non-specific factors. PD-specific risk factors for anxiety include younger age at onset^14,15^ motor fluctuations,^14–16^ shorter disease duration^16^ faster rate of progression^14^ greater disease severity^14,15^ postural instability gait disorder subtype^15,17^ dysautonomic features^18^ REM sleep behavior disorder^18^ and larger echogenic areas in the substantia nigra^18^ GAD and mixed anxiety in PD have been linked to younger age.^14,16^

Non-specific risk factors include female gender,^4,16,18,19^ history of anxiety^16^ and depression^12,13,18^ and family history of psychiatric disorders.^12,13^ Psychological risk factors have also been implicated. Reliance on emotion-focused, as opposed to problem-focused coping skills, perception of loss of internal and external control over disease symptoms, less social support and avoidant personality traits, have been associated with increased symptoms of anxiety.^20^

The association between anxiety and motor fluctuations, the significant exacerbation of anxiety provoked by dopamine withdrawal, suggests that neurodegeneration of the dopaminergic pathways is involved in anxiety in PD. This is also supported by a recent study showing that in newly-diagnosed, drug naïve patients, lower DA uptake at the caudate was associated with more severe trait anxiety.^21^ However, increasing L-dopa has not been shown to reliably reduce anxiety in treated PWP. The pathophysiology of PD-related anxiety disorders is complex and remains to be elucidated. In rats, the unilateral 6-hydroxydopamine (6-OHDA) lesion of the medial forebrain bundle induces decreased dopamine levels in the limbic regions (striatum, medial prefrontal cortex, amygdala, and hippocampus) and causes anxiety-like behaviors.^22^ It also disrupts the serotoninergic modulation of the neurons of the amygdala.^22–24^ This imbalance between DA and 5HT activity might be an etiological and pathophysiological factor for anxiety in PD.^24^ This was also suggested by studies in monkeys^25^ and in early-diagnosed drug-naïve PD patients.^26^ In PD, hypofunction of the amygdala might play a key role in the development of anxiety disorders.^27^ The basolateral amygdala is a densely connected “hub” relaying sensory information to the central amygdala which is the major efferent pathway with outputs namely to the frontal cortex (representation of fear attributes) and the hippocampus (memory of fear). However, although the role of the amygdala in anxiety and fear has been well studied in the general population, there is still controversy and we are unsure how it can be applied to PD. For instance, it is debated whether the amygdala is centrally responsible for both the autonomic/behavioral reaction to anxiety and the cognitive-emotional experience (‘central fear generator’ hypothesis) or part of a ‘two-system’ framework that requires additional circuitry for the autonomic/behavioral response. (as reviewed in Fanselow MS and Pennington ZT 2018).

So far, only twelve studies have been published. Most were PET or SPECT studies and only three were volumetric studies.^21,28–38^ Overall, the functional studies of anxiety in PD showed involvement of the striatum and of the DA and 5HT pathways. The anatomical studies showed reductions in the volume of several brain areas, namely the amygdala, the anterior cingulate cortex and the orbito-frontal cortex. Recent connectivity studies showed that anxiety in PD was related to changes in the striatum-frontal cortex network.^39,40^ Hence, key structures in the processing of emotions in general and in the neurocircuitry of fear, in particular, seem to be involved.^41^ However, all these studies, except one,^37^ were correlation studies in PWP without established anxiety disorders, meaning the changes might be due to factors other than the presence or absence of anxiety. Moreover, anxiety measures were not always optimal, namely many used anxiety scales for which there is a high overlap between symptoms of anxiety and those of PD. Further studies directly comparing PWP with and without anxiety are thus needed. Moreover, as all these regions are part of a large network, additional studies using innovative structural and functional connectivity approaches are required.

Given the high prevalence and adverse impact of anxiety syndromes in PWP the use of an anxiety rating scale may be helpful for screening, determining the severity of symptoms, and to quantify effects of treatment.

Whereas depression rating scales have been validated and used for decades in PD,^42^ a Movement Disorder Society (MDS) task force concluded in 2008 that none of the available anxiety rating scales were validated for use in PD.^43^ The first validation study of the Beck Anxiety Inventory (BAI), the Hamilton Anxiety Rating Scale (HAMA), and the Hospital Anxiety and Depression Scale (HADS) was done by an international consortium in 2011. This consortium reported that while some clinimetric properties of these scales are acceptable, such as ease of completion (e.g., acceptability), known groups validity, inter-rater reliability and test-retest reliability, there are also important shortcomings such as the limited construct validity, the low convergent validity with other anxiety rating scales, as well as low divergent validity with depression rating scales. The latter implies that these scales cannot separate depression from anxiety.^44^ None of the existing scales assessed avoidance behavior, and they generally lacked sensitivity in the lower score ranges (i.e., floor effect), leading to under recognition of anxiety. Another study validated the Geriatric Anxiety Inventory (GAI) in PWP, with assessment of a limited number of clinimetric properties. The GAI showed good internal consistency and test-retest reliability.^45^

The development and validation of the Parkinson Anxiety Scale (PAS) by the same international consortium was intended to overcome the clinimetric shortcomings of the existing anxiety rating scales in PD.^46^ This consortium followed a strictly systematic approach to the development of the scale. The general format of the scale was formulated using the Delphi procedure. Sections of the scale were defined based on clinical presentations of anxiety using latent class analysis. Item selection was based on cannonical correlation analysis performed using existing rating scales and the database of the earlier validation study.^47,48^ The PAS is divided into three sections that assess episodic anxiety, persistent anxiety, and avoidance behavior. Clinimetric properties are superior to those of other anxiety rating scales, with satisfactory acceptability, distribution across score range, internal consistency, test-retest reliability, inter-rater reliability, sensitivity, and specificity. The scale has been translated in many languages.

However, there is still room for improvement. Those anxiety rating scales that have been validated have all been studied in PWP without dementia. Validation of these scales in PWP with dementia remains to be done.^49^ Moreover, anxiety scales specifically developed for patients with dementia in the general population still need to be validated in the context of PD dementia, such as the rating anxiety in dementia (RAID) scale. A shortcoming of the PAS, as well as other anxiety scales, is that they assess only common presentations of anxiety. Some presentations of anxiety that are unique to PD are not assessed, such as anxiety during ‘wearing-off’ of medication, anxiety during ‘off’ periods, and situational anxiety e.g. during the performance of activities of daily living that present a risk of falling.^49,50^ For this reason the development of a PD-specific anxiety questionnaire has been proposed. A preliminary study profiled 30 triggers of PD-related anxiety. The presence of distress, worry, fear, agitation, embarrassment, and avoidance were frequently reported as resulting from motor symptoms including complications of medication for PD.^11^ This study will be the basis for the development of a new inventory to assess PD specific triggers of anxiety, and resulting behavior, in both research settings and clinical practice, named the PD Specific Anxiety Inventory (PDSAI).

PWP are particularly vulnerable to side effects of some of the most commonly-used pharmacological treatments of anxiety, such as increased risk of falls and cognitive impairment in the case of benzodiazepines or antihistamines.^51^ Therefore, there is a need for non-pharmacological approaches, including behavioral interventions and neurostimulation. PWP have a high level of willingness to participate in behavioral interventions for their illness.^52^ The ideal behavioral intervention for PD would have the following attributes: (1) it would be effective in reducing anxiety as well as depression, given their frequent co-occurrence; (2) it would improve or even prevent the decline in cognitive function associated with PD; (3) it would be accessible in the community. Physical exercise has few adequately powered trials to guide its use for anxiety in PD but is of interest because of its overall benefit to the motor symptoms of the disease. A recent RCT of 138 PWP compared a mindfulness yoga program to a stretching and resistance training exercise and found that the mindfulness yoga program produced a comparable improvement in motor symptoms with the additional benefit of reducing anxiety and depression.^53^ This suggests that ‘mind-body’ exercises may be one of the most promising ways to meet the ideals stated above. Psychotherapy and psychosocial interventions are known to be effective for anxiety in general but only few studies have assessed their effectiveness in PD.^54^

Cognitive behavioral therapy (CBT) is considered the gold standard of psychotherapy treatments for anxiety. In PD anxiety, only a few single-case or open label studies have been reported. Overall, studies were limited by methodological weaknesses. They were underpowered, enrolled patients with mixed depression and anxiety symptoms and had variable results. Some studies showed a significant reduction of depression and anxiety symptoms in the CBT group compared with controls^55^ while others reported a benefit on depression but no effect on anxiety.^56^ Moreover, long term effects were also variable. More powerful, randomized, controlled trials focused on the effects of CBT on anxiety are thus needed. Some are in progress and their results will probably provide guidelines on the best way to administer such therapy in PWP with anxiety disorders.^57^

Mindfulness training has the potential for managing a variety of neuropsychiatric conditions, including anxiety disorders. While the concept of mindfulness is thousands of years old, protocol driven mindfulness training courses have increased its presence in Western medicine. Exemplary of this approach is mindfulness-based stress reduction, or MBSR, a group-based 8-week introduction to mindfulness meditation that was created at University of Massachusetts in the 1980s and is available throughout the US and worldwide. MBSR is not specific to PD, but may be helpful for both anxiety and cognitive functioning in older adults.^58^ No published studies have demonstrated the effectiveness of mindfulness training in PD, although several pilot studies are ongoing and preliminary findings are supportive.^59–61^ Another advantage of mindfulness training is the potential to deliver it remotely, via mHealth/telehealth, which may be more accessible to PD populations with limited mobility.^62^ At this time, the evidence for mindfulness training in PD is inconclusive;^63^ more research is needed to test its effectiveness and innovative ways to deliver it into the community.

Another approach that deserves study is to specifically target some of the cognitive biases (e.g., biased attention to stimuli signaling threat, or biases towards negative interpretations of ambiguous situations). Cognitive bias modification interventions are showing promise in the management of a range of anxiety disorders including generalized anxiety,^64^ but have yet to be systematically explored in PD. Such computer-based interventions, if effective, have the potential to be simple, accessible and economical ways to deliver interventions to people in their own homes.

Non-invasive brain stimulation (NIBS) such as repetitive transcranial magnetic stimulation (rTMS), and transcranial direct current stimulation (tDCS) in the treatment of anxiety is still in its infancy. rTMS is a non-invasive procedure in which cerebral electrical activity is modulated by a rapidly changing magnetic field. Outside the context of PD, there is some growing evidence that GAD and panic disorders might benefit from rTMS,^65^ and that anxiety can be reduced in patients with major depressive disorders.^66^ In PD, few studies addressed the effect of NIBS on anxiety. None of them had anxiety as a primary outcome. One open label study in PD patients with treatment-resistant depression found a significant improvement on both depression and anxiety following multiple sessions of 10 Hz rTMS over the right dorsolateral prefrontal cortex (DLPFC) up to 6 weeks after treatment.^67^ Traditional tDCS delivers a constant low direct current through an anodal and a cathodal electrode on the head. In a study evaluating the impact of anodal tDCS over the left vs. the right DLPFC on executive function in PD, a significant post-treatment effect of left DLPFC stimulation vs. right and sham tDCS stimulation was found in PD patients for depression but not for anxiety.^68^ Apart from the fact that it is well tolerated, little inference based on the current evidence can be made regarding the use of NIBS as treatment for anxiety in PD. Additional large-scale, randomized studies are warranted in PWP with moderate to severe anxiety to determine the optimal protocols with respect to stimulation parameters, timing and number of sessions.

Deep brain stimulation (DBS) is a neurosurgical procedure which sends electrical impulses, through implanted electrodes, to specific targets in the brain such as the subthalamic nucleus (STN) and the internal global pallidus (GPi) for motor symptoms. Several studies have investigated its effect on anxiety, more so for STN stimulation than GPi. While results are heterogenous, at the group level improvement of anxiety is apparent after DBS and more pronounced in the short-term.^69^ However, some patients experience transient, clinically significant neuropsychiatric symptoms including anxiety following DBS. These have been noted on occasion after the reduction of dopamine therapy following STN DBS with improvement after increasing it.^70^ A recent study indicates that the exact placement of the electrode in the STN may impact the various non-motor symptoms differently, suggesting it may be possible to personalize DBS therapy by tailoring it to the specific motor and non-motor symptom profile including anxiety.^71^

There is thus a real need for additional studies on such interventions targeting PD-related anxiety.

In contrast to treatment for depression, to date there is little trial evidence for the pharmacological treatment of anxiety. Whilst off-periods symptoms often include severe anxiety which improves with dopaminergic medication,^72,73^ and anxiety is frequently part of the dopamine agonist withdrawal syndrome, there have been no randomized, placebo-controlled, clinical trials of medication for the treatment of anxiety in PD. Clinicians most commonly prescribe selective serotonin reuptake inhibitors (SSRIs) and benzodiazepines. However, in the multicenter Study of Antidepressants in Parkinson’s Disease (SAD-PD) trial both paroxetine (an SSRI) and venlafaxine XR (a serotonin norepinephrine reuptake inhibitor or SNRI) were more effective than placebo for the treatment of depressive symptoms, but neither demonstrated any effect on a secondary anxiety measure.^74^ In fact, higher baseline anxiety levels predicted poorer response to treatment of depressive symptoms.^75^ A trial comparing desipramine and citalopram with placebo for depression also did not show a greater improvement of anxiety scores on these treatments than on placebo. Another randomized placebo-controlled trial comparing nortriptyline and paroxetine for the treatment of depression in PD demonstrated no effect for paroxetine on a secondary anxiety measure.^76^ In a trial of atomoxetine for the treatment of depression, there was a non-significant suggestion for greater improvement of anxiety as a secondary outcome measure compared to placebo.^77^ However, none of these studies was designed to detect changes in anxiety, so they highlight the need to further evaluate treatment approaches for anxiety in PD. Benzodiazepine therapy is not ideal given a side effect profile including sedation, cognitive impairment or delirium, and balance impairment associated with an increased risk of falls. Long term use may also be associated with tolerance and increased risk of dementia.^78^

There is currently a placebo-controlled, double-blind, pilot study of buspirone in 27 participants with PD and anxiety underway to test the tolerability of buspirone, a 5HT1A receptor partial agonist with weak dopamine D2 and 5HT2 receptor activity,^79^ which has been shown to be effective for the treatment of GAD.^80–82^ It has been shown to be beneficial in PD in two small trials, but through its effects on the dopaminergic system and weak affinity for D2 receptors could potentially be associated with worsening of PD motor function. In addition, a large, placebo-controlled trial of the antidepressants escitalopram and nortriptyline (Adept-PD) will assess the impact of these medications on depression and anxiety in patients with depression in PD in the UK.

Integrated care, also known as inter-disciplinary or multidisciplinary (“multi-D”) care, coordinated care, comprehensive care, seamless care, or transmural care, is being adopted worldwide in a variety of specialties and health care areas. There is an ongoing process with reforms and new organizational arrangements focusing on more coordinated and integrated forms of care provision. Integrated care may be seen as a response to the fragmented delivery of health and social services being an acknowledged problem in many health systems.^83–85^ Integrated care represents the highest level in the continuum of autonomy to cooperation to integration.^86^

Integrated health care models have been found to be efficacious compared to non-integrated delivery in decreasing depression and anxiety scores in the elderly in general^87^ and there is evidence for the benefit of multidisciplinary care for neuropsychiatric symptoms specifically in PD.^88^ Further, mindfulness-based stress reduction showed positive patient-centered outcomes and favorable health care utilization in an integrated health care delivery system.^89^

The implementation of integrated or multidisciplinary care in anxiety requires the reorganization of the current fragmented care delivery setup, which is based on autonomy. It is necessary to develop clinical pathways and instruction materials. Effective flow of information and active multi-directional coordination is usually achieved in the form of regular multidisciplinary meetings and continuous electronic and optional occasional phone/personal communications. Education and outreach are often a part of integrated health care models. It is necessary to adopt formal quality control metrics, which can be part of the meetings or dedicated “circles” specifically for this purpose as part of the implementation process. Implementation is often impeded due to the current reimbursement systems and training curricula for health care professionals.^90,91^ Despite these challenges, there is evidence for the benefits of integrated care in mental health,^86^ specifically in neuropsychiatric symptoms (such as anxiety) in PD,^88^ and supported by anxiety patients’ preference for integrated care.^92^

Caregivers play an integral part in meeting the physical, emotional, and financial needs of PWP and are critical members of the healthcare team. Caregiving in PD is predominantly provided by informal caregivers, mostly spouses. Estimates suggest that caregivers assist PWP an average of 11 times per day in early PD and up to 30 times per day in later stages of the disease.^93^ Unfortunately, the majority of PD caregivers experience burden in the context of their helping role, which may be higher than that endured by caregivers in other medical populations.^94^ Non-motor symptoms in PWP, such as anxiety, have consistently been found to be one of the strongest predictors of PD caregiver stress.^95^

Caregiver burden may increase the risk that caregivers will engage in negative social exchanges with PWP, exacerbating the PWPs’ symptoms of both PD and anxiety. Moreover, caregiver burden is significantly associated with the caregivers’ poorer recognition and understanding of both anxiety and depression in PWP,^96^ negatively influencing the patient-caregiver dyadic relationship. Conversely, positive social support has been associated with improved coping, as well as decreased disability, depression, and anxiety in the PD population, even when controlling for disease severity.^97^ Importantly, structured caregiver involvement in CBT interventions for depression in PD, was the strongest predictor of patient treatment response, accounting for more than 25% of the variance in improvement trajectories.^98^

Improving anxiety in PD, and the integral relationship with the caregiver can potentially be addressed in following ways: (i) patient interventions to address non-motor symptoms and increase independence in PWP, (ii) caregiver specific interventions to treat caregiver psychological symptoms (i.e., referral for individual counselling), and (iii) patient-caregiver dyadic interventions. Given the reciprocal relationship between patient and caregiver health, unique clinical considerations for caregiver involvement in the treatment of anxiety in PD must be critically evaluated on a case-by-case basis to best meet the needs of each dyad. Factors such as the nature of the relationship between the PWP and carepartner (spouse, child), quality and characteristics of the relationship (active benefit-finding in the caregiver role vs. anger and burden), and specific type of caregiver intervention which may be most appropriate require careful consideration. Dyadic intervention can be delivered in the form of marital/ family interventions,^99^ parallel interventions,^100^ or partner-assisted interventions.^101^ For example, in a stable marital relationship characterized by the reciprocal exchange of support, a partner assisted intervention for anxiety in PD could be quite beneficial. A partner assisted CBT intervention for anxiety in PD was successfully completed in a recent study.^101^ In this intervention, education about anxiety in PD was delivered to both PD and their carepartners, and the carepartner was included to informally coach the PWP as they practiced the newly acquired CBT coping skills learned throughout treatment (e.g., relaxation, meditation, “talking back” to negative thoughts, exercise) in the home environment. Caregivers were also asked to assist in the PWP proactive engagement in their own lives and self-care, rather than giving in to the withdrawal and avoidance behavior that commonly characterizes anxiety in PD. The CBT intervention was associated with significantly reduced anxiety immediately post-intervention, with sustained reductions in anxiety at 6-month follow-up, as well as significantly decreased caregiver burden at post intervention.

Compared with depression, anxiety in PD has received less overall attention to date (in part due to its significant overlap with depression), although that has changed in recent years. Cross-sectional studies have documented that up to 40% of PWP experience anxiety symptoms or disorders, including GAD, panic attacks, and agoraphobia or social phobia.^12,15,102,103^ Increasing anxiety and discrete anxiety attacks have been associated with non-motor fluctuations in patients with more advanced PD and chronic exposure to higher-dose levodopa treatment, maybe more so at the onset of “off” periods.^12,15^ However, anxiety in PD is not simply a late disease manifestation, as there is an increased frequency of anxiety disorders up to 20 years prior to PD diagnosis on the basis of motor symptoms,^4,104^ providing biological plausibility.

Advances have been made in the assessment of anxiety with the recent development and validation of a PD-specific anxiety rating scale (the PAS).^46^ A recently developed neuropsychiatric fluctuations scale includes a number of anxiety items,^105^ and the new movement disorders society-non-motor rating scale (MDS-NMS), which is currently being validated, includes four anxiety questions and a separate non-motor fluctuations section with an anxiety item.

Overall, relatively little is known about the etiology or neurobiology of anxiety in PD. There have also been scant pharmacological treatment studies for anxiety in PD and the recent International Parkinson and MDS evidence-based review for the treatment of non-motor symptoms of PD found that there were no randomized clinical trials (RCTs) for the treatment of anxiety disorders that met inclusion criteria.^106^ Antidepressant treatment studies, in general, have not reported secondary benefit for anxiety symptoms. For patients who experience anxiety as part of an “off” state, PD medication adjustments can be made in an attempt to decrease the duration and severity of these episodes. However, some patients require treatment with benzodiazepines, although this medication class must be used cautiously in PWP due to their propensity to increase sedation, gait imbalance, and cognitive impairment.

Ongoing, longitudinal, relatively large epidemiological studies will improve our understanding of the evolution (e.g., cumulative prevalence, incidence) and predictors of anxiety symptoms over the course of PD. These studies either enroll de novo, untreated PD patients at baseline (e.g., PPMI, DeNoPa, and ICICLE-PD) or established PD patients (DEMPARK/LANDSCAPE), both of which are informative. These studies typically include some multi-modal neurobiological assessments, which will help improve our understanding of the neural substrate in PD.

In terms of treatment, there are ongoing RCTs of pharmacological (e.g., rotigotine patch and buspirone) and non-pharmacological (cognitive-behavioral therapy and mindfulness-based stress reduction) treatments for anxiety in PD. There are also clinical trials examining the treatment of non-motor symptoms, including anxiety, broadly (e.g., levodopa-carbidopa intestinal gel in advanced PD). Lacking are any RCTs examining the efficacy and tolerability of commonly-used antidepressants or benzodiazepines for anxiety in PD, and in the future, there will likely be studies examining the effects of medical marijuana for this condition.

## Discussion

Of the many things that go wrong in PD, anxiety is certainly near the top of the list of the neurobehavioral problems because of the distress it causes and the prevalence. However, the number of published studies has been impressively and embarrassingly small. Until the 1980s, neurobehavioral studies focused on depression, and, although depression and anxiety go hand in hand, little attention was paid to anxiety. Anxiety in PD differs in a few ways from that in the general population, affecting the genders about equally, developing much later in life. A common misconception is that it is a response to the disease or embarrassment due to motor symptoms in social situations. However, risk factors include young age at onset, more rapid progression, more severe motor dysfunction, and motor fluctuations—suggesting a biological link to PD. We don’t yet know the natural history. Does it worsen, resolve or fluctuate over time? Does it presage other neurobehavioral problems? One problem has been the lack of validated scales, especially one validated in patients with dementia. In the future remote monitoring of anxiety in real time using at-home or mobile technology is likely to provide a more reliable assessment of the phenomenology of anxiety (including more objective signs such as heart rate variability) and triggers that historical ‘out-of-anxious-state’ assessment cannot provide. To ensure the replication and generalizability of conclusions from existing and future work on anxiety in PD, standardized assessment and consistent methodology are needed.

We have rudimentary data on neurotransmitter and physiological changes associated with anxiety, based primarily on imaging studies and animal observations. These implicate both dopamine and serotonin, but how important this information will be to developing an understanding and treatment remains unknown. And, as a result of this weak data base, our drug therapies are based almost entirely on observations in the general population.

Perhaps, almost incredibly, there have been no published trials of medication treatment of anxiety. The only pharmacologic data are based on the few antidepressant trials, where anxiety was a secondary variable and the samples too small to infer results. Non-pharmacologic therapies, e.g., CBT, have been proven helpful but have not been widely embraced and access will remain logistically difficult until we become more comfortable with telemedicine. The participants universally endorsed a multidisciplinary approach to treating anxiety, including neurologists, psychiatrists and other mental health care workers, as well as support personnel. Special attention is needed for caregivers as well, since they are the front line of support. Sometimes treating a caregiver may be more important than treating the patient.

Future work needs to focus on all aspects of anxiety: defining and validating measures in both demented and non-demented patients; understanding its pathophysiology, probably through imaging and possibly with biomarkers; monitoring its natural history; developing drug treatments and improving non-drug treatment. In the mean-while it is important to educate our patients, their support systems and our colleagues, and try to develop cost effective multidisciplinary models of care.
