# Author Correction: Report from a multidisciplinary meeting on anxiety as a non-motor manifestation of Parkinson’s disease

**DOI:** 10.1038/s41531-020-0114-4

**Published:** 2020-06-02

**Authors:** Gregory M. Pontone, Nadeeka Dissanayaka, Liana Apostolova, Richard G. Brown, Roseanne Dobkin, Kathy Dujardin, Joseph H. Friedman, Albert F. G. Leentjens, Eric J. Lenze, Laura Marsh, Lynda Mari, Oury Monchi, Irene H. Richard, Anette Schrag, Antonio P. Strafella, Beth Vernaleo, Daniel Weintraub, Zoltan Mari

**Affiliations:** 1 Department of Psychiatry and Behavioral Sciences, Johns Hopkins University School of Medicine, Baltimore, MD USA; 2 Department of Neurology, Johns Hopkins University School of Medicine, Baltimore, MD USA; 3 The University of Queensland Centre for Clinical Research, Faculty of Medicine, Brisbane, Australia; 4 School of Psychology, The University of Queensland, Brisbane, Australia; 5 Department of Neurology, Royal Brisbane & Woman’s Hospital, Brisbane, Australia; 6 Department of Neurology, Indiana University School of Medicine, Indianapolis, IN USA; 7 Department of Psychology, Institute of Psychiatry Psychology and Neuroscience, King’s College London, London, UK; 8 South London and Maudsley NHS Foundation Trust, London, UK; 9 Department of Psychiatry, Rutgers University, Robert Wood Johnson Medical School, Piscataway, NJ USA; 10 Department of Neurology and Movement Disorders, Lille University Medical Center, Lille, France; 11 Movement Disorders Program, Butler Hospital; Department of Neurology, Warren Alpert Medical School of Brown University, Providence, RI USA; 12 Department of Psychiatry, Maastricht University Medical Center, Maastricht, the Netherlands; 13 Department of Psychiatry, Washington University School of Medicine, St. Louis, MO USA; 14 Michael E. DeBakey Veterans Affairs Medical Center, Houston, TX USA; 15 Department of Psychiatry, Baylor College of Medicine, Houston, TX USA; 16 Person Holistic Innovation, Las Vegas, NV USA; 17 Departments of Clinical Neurosciences and Radiology, Hotchkiss Brain Institute, University of Calgary, Calgary, Canada; 18 Department of Neurology, University of Rochester Medical Center, Rochester, NY USA; 19 Department of Clinical and Movement Neurosciences, University College London, London, UK; 20 E.J. Safra Parkinson Disease Program, Toronto Western Hospital & Krembil Research Institute, UHN; Research Imaging Centre, Campbell Family Mental Health Research Institute, CAMH, University of Toronto, Ontario, Canada; 21 Parkinson’s Foundation, New York, NY USA; 22 Departments of Psychiatry and Neurology, Perelman School of Medicine at the University of Pennsylvania, Philadelphia, PA USA; 23 Parkinson’s Disease Research, Education and Clinical Center, Philadelphia Veterans Affairs Medical Center, Philadelphia, PA USA; 24 Cleveland Clinic Lou Ruvo Center for Brain Health, Movement Disorders Program, Las Vegas, NV USA

**Keywords:** Parkinson's disease, Parkinson's disease

Correction to: *npj Parkinson’s Disease* 10.1038/s41531-019-0102-8, published online 11 December 2019

The original version of the published Article contained an error in the spelling of the second Author’s name. “Nadeeka Dissanayka” has been changed to “Nadeeka Dissanayaka”. This has been corrected in the HTML and PDF version of the Article.

